# Magnetic Resonance Imaging of Phosphocreatine and Determination of BOLD Kinetics in Lower Extremity Muscles using a Dual-Frequency Coil Array

**DOI:** 10.1038/srep30568

**Published:** 2016-07-28

**Authors:** Ryan Brown, Oleksandr Khegai, Prodromos Parasoglou

**Affiliations:** 1 Bernard and Irene Schwartz Center for Biomedical Imaging, Department of Radiology, New York University School of Medicine, New York, NY, USA; 2 Center for Advanced Imaging Innovation and Research (CAI2R), Department of Radiology, New York University School of Medicine, New York, NY, USA; 3 NYU WIRELESS, Polytechnic Institute of New York University, 2 Metro Tech Center, Brooklyn, NY, 11201, USA.

## Abstract

Magnetic resonance imaging (MRI) provides the unique ability to study metabolic and microvasculature functions in skeletal muscle using phosphorus and proton measurements. However, the low sensitivity of these techniques can make it difficult to capture dynamic muscle activity due to the temporal resolution required for kinetic measurements during and after exercise tasks. Here, we report the design of a dual-nuclei coil array that enables proton and phosphorus MRI of the human lower extremities with high spatial and temporal resolution. We developed an array with whole-volume coverage of the calf and a phosphorus signal-to-noise ratio of more than double that of a birdcage coil in the gastrocnemius muscles. This enabled the local assessment of phosphocreatine recovery kinetics following a plantar flexion exercise using an efficient sampling scheme with a 6 s temporal resolution. The integrated proton array demonstrated image quality approximately equal to that of a clinical state-of-the-art knee coil, which enabled fat quantification and dynamic blood oxygen level-dependent measurements that reflect microvasculature function. The developed array and time-efficient pulse sequences were combined to create a localized assessment of calf metabolism using phosphorus measurements and vasculature function using proton measurements, which could provide new insights into muscle function.

*In vivo* phosphorus magnetic resonance spectroscopy (^31^P-MRS) has been used extensively to assess energy metabolism in many diseases that affect skeletal muscle^1^. ^31^P-MRS can measure phosphocreatine (PCr) and inorganic phosphate (Pi) kinetics and yield valuable information about muscle mitochondrial function during exercise^2^,^3^,^4^.

Due to the low concentration of phosphorus-containing metabolites in human tissue^5^ and the low intrinsic sensitivity of ^31^P-MR, most ^31^P-MRS studies are performed using small surface coils that are placed adjacent to the muscle of interest^4^. Such coils can provide an adequate signal-to-noise ratio (SNR) in a local region, but they exhibit an inhomogeneous magnetic field and limited coverage. These characteristics typically require lengthy power-demanding adiabatic pulses to generate an excitation field with adequate uniformity and prohibit simultaneous analysis of metabolic activity in multiple lower extremity muscles^6^,^7^,^8^. Meanwhile, birdcage coils have been used to acquire whole-volume ^31^P-MR data with a homogeneous field distribution but with a reduced SNR compared to surface coils, which can result in compromised spatial-temporal resolution and/or acquisition time^9^,^10^.

^31^P-MR exercise protocols require suitable temporal resolution to adequately capture mitochondrial function. Previously, we demonstrated a protocol with a birdcage coil at 3 Tesla that provided volumetric measurements of the lower leg with a 24 s temporal resolution^11^. This resolution was reduced to 12 s by combining the SNR advantage at 7 Tesla with an efficient compressed sensing data acquisition scheme^12^. However, this rate can make it challenging to accurately model PCr recovery following exercise^13^, and it is significantly slower than the rate achieved in studies performed with surface coils and unlocalized spectroscopy sequences (typically 6 s)^14^.

Recently, phased array coils have been designed to combine the SNR of surface coils with the volumetric coverage of birdcage coils for low SNR, non-proton applications^15^,^16^,^17^,^18^,^19^,^20^,^21^,^22^,^23^,^24^,^25^,^26^,^27^. In this study, we developed and evaluated a ^31^P/^1^H array for lower extremity MRI. The array consisted of an encompassing eight-channel ^31^P module to provide volumetric coverage of the lower extremities along with a high SNR that can be exchanged for temporal resolution. Additionally, the developed array was equipped with an integrated eight-channel ^1^H module that enabled clinical-quality MRI measurements, which is essential for assessing micro- and macro-vascular functions in skeletal muscle using dynamic techniques such as blood oxygenation level dependent (BOLD) MRI^28^ and arterial spin labeling (ASL)^29^. We generated benchmark performance measurements to compare the developed array to commercially available alternatives. Preliminary *in vivo* experiments additionally demonstrate the results of volumetric ^31^P and ^1^H imaging with high spatial and temporal resolution during lower extremity exercise protocols.

## Results

### ^31^P benchmark measurements

**Figure 1:**
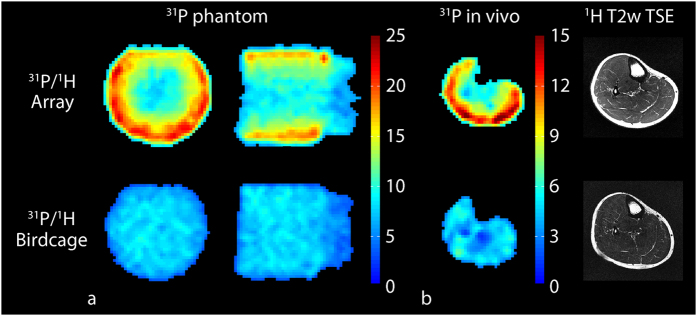
Characterization of the eight-channel ^31^P module of the developed array. The array provided a similar ^31^P SNR in the center and greater than 2-fold gain in the periphery over the birdcage in the 42 mM Pi phantom (**a**). Similar results were observed *in vivo* (**b**). The measurements are summarized in Table 1.

*In vivo* measurements showed that the SNR provided by the ^31^P module of the developed eight-channel array was more than 2.4 times greater than the SNR of the commercially available single-channel reference volume coil (birdcage) in peripheral muscles such as the medial and lateral gastrocnemius, which are recruited for pedal flexion (see Fig. 1 and Table 1). The SNR gain in locations near the center of the calf, such as the tibialis posterior, were in the range of 30%. The transmission efficiencies for spin excitation in the central transverse plane of a water phantom (15 cm diameter, 2 L, doped with 42 mM Pi) were 253.4 ± 14.3 and 323.2 ± 6.5 nT/V for the developed array and reference coil, respectively. The ~30% reduction in transmission efficiency compared to the reference coil was partially due to losses in the multi-stage power divider that is required in the interface chain as well as inter-element coupling. Both losses were considered to be tolerable consequences of the multi-element transmission configuration.

### ^1^H benchmark measurements

**Figure 2:**
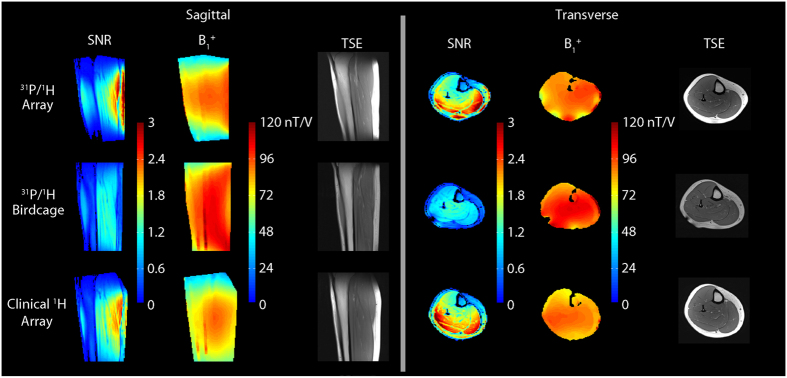
Normalized ^1^H SNR maps (first and fourth columns), B_1_^+^ maps (second and fifth columns), and TSE images (third and last columns) acquired with the eight-channel ^1^H module (top row) of the developed array. The array showed favorable performance over the dual-frequency birdcage coil (middle) and similar performance to the clinical 15-channel ^1^H array (bottom). The measurements are summarized in Table 2.

The ^1^H SNR provided by the developed dual-nuclei array was superior to the dual-nuclei volume coil (approximately 2.4 times greater in the gastrocnemius muscles) and similar to (within 15%) that of a state-of-the-art 15-channel mono-nuclear clinical array. The ^1^H transmission efficiency and uniformity were similar for all three coils (see Fig. 2 and Table 2). The spin echo anatomical images exhibited good quality with no signs of artifacts.

### Dynamic ^31^P-MRI

**Figure 3:**
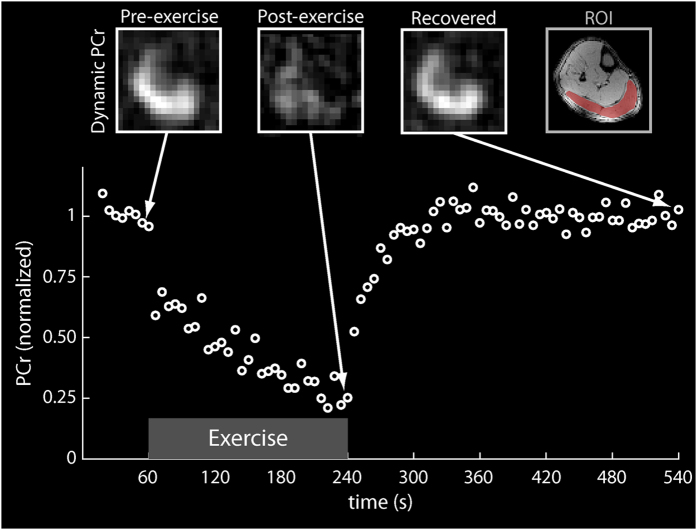
Dynamic ^31^P PCr (top row) images acquired using the developed coil array in the calf muscle at different time points during the plantar flexion exercise protocol with the PCr signal time course (bottom) from the segmented gastrocnemius muscle (top right).

Figure 3 shows dynamic imaging of PCr using a spectrally selective ^31^P-FLORET pulse sequence with a 6-s temporal resolution. The time series SNR of the PCr signal from the gastrocnemius muscle at rest was 19.0. A 30-year-old male participant performed plantar flexions at 0.33 Hz according to an acoustic cue using an MR compatible ergometer that was built in-house^30^. The PCr signal in the gastrocnemius muscle was fit to a single exponential recovery function to determine PCr depletion (77%) and the PCr resynthesis rate (*k*_PCr_ = 20.5 s, *r*^*2*^ = 0.91).

### ^1^H-MRI

**Figure 4:**
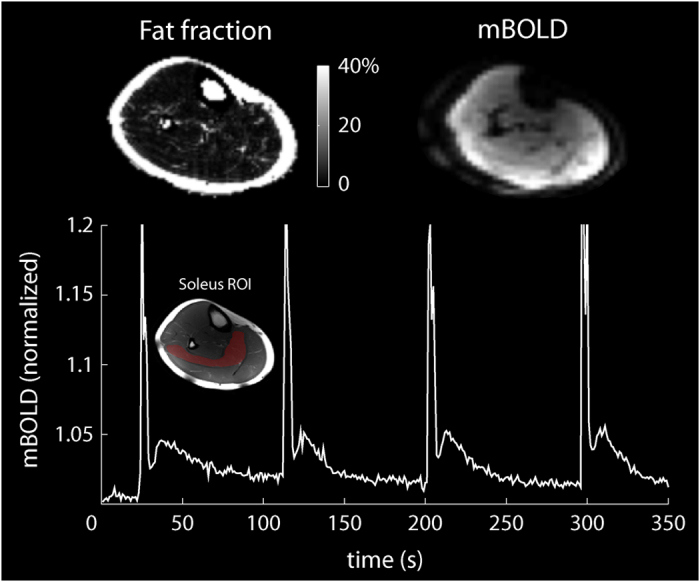
*In vivo* fat fraction and mBOLD images (top row) and mBOLD signal evolution in the soleus muscle (bottom row, ROI inset) following maximum voluntary isometric plantar flexions.

Many diseases that are known to affect skeletal muscle metabolism, such as obesity, diabetes, and muscular dystrophy, also result in fat infiltration into the muscle^31^. To demonstrate the ^1^H-MRI capability of the coil, we calculated water and fat fraction maps using the Hierarchical IDEAL method^32^ from data acquired with a multi-echo three-dimensional gradient echo sequence in a 31-year-old male volunteer with a BMI of 25.5 (see Fig. 4). Low fat infiltration (<2.5%) was observed in all muscle groups of the lower leg in this subject.

BOLD changes in the soleus muscle were measured following voluntary maximal isometric plantar flexion contractions using a one-shot gradient-recalled echo-planar sequence. We acquired images during a 10 min period while the subject performed 1-s isometric contractions every 90 s. The spikes in the BOLD signal during the contractions were followed by delayed transient signal increases (see Fig. 4). The relative BOLD signal increase (ΔS_max_) was 2.9%, while the time-to-peak (TTP) was 12.5 s.

## Discussion

In this study, we demonstrated that an eight-channel ^31^P transmit/receive RF array paired with a “nested” eight-channel ^1^H array could provide high-quality ^31^P and clinical-quality ^1^H data on a clinical 3 Tesla MR device. We combined the coil with efficient pulse sequences, (i.e., ^31^P-FLORET) to measure PCr kinetics following exercise with a 6-s temporal resolution. To the best of our knowledge, this temporal resolution is the highest achieved with a volumetric imaging pulse sequence, and it is comparable to the resolution obtained using unlocalized spectroscopy^11^,^12^,^33^.

The essential goal of this project was to develop a coil that facilitates acquisition of high-quality ^31^P and ^1^H MR data without the need to reposition the subject, which is required when two separate mono-nuclear coils are used. Our array provided a substantial ^31^P SNR gain over a conventional birdcage coil. The ^31^P structure was free of the SNR-lowering circuitry that may be found in dual-nuclei coils, such as trap circuits or in-line positive-intrinsic-negative (PIN) diodes^34^,^35^. The proposed approach resulted in ^31^P SNR gains of 1.3- to 2.4-fold *in vivo*. By combining the ^31^P transmit and receive functionalities into a single structure, a separate transmit coil was eliminated as well as the lossy components, such as fuses and detuning circuits, that are required in receive-only coils. In transmit mode, we drove the structure with circular polarization to generate an excitation field with a uniformity similar to that of the reference birdcage, which may eliminate the need for adiabatic excitation pulses that can be very lengthy, particularly when inverting deep lying spins. Our array additionally provided ^1^H imaging and B_0_ shimming capabilities with performance that was similar to that of a clinical device. This development enabled us to create comprehensive multinuclear MR protocols that can minimize experimental variability by avoiding subject repositioning, which reduces scan time^36^, as well as enable interleaved or simultaneous multinuclear acquisition using specialized pulse sequences and back-end RF hardware^33^,^37^,^38^.

In summary, our ^31^P/^1^H array offers a unique ability to investigate several aspects of muscle function, including regional perfusion^39^, blood tissue oxygenation through ^1^H-MR^28^,^40^, and intracellular pH and mitochondrial function^4^ through ^31^P-MR, in healthy and disease states. Relatively high spatial/temporal resolution multi-nuclear MR can be performed over the entire calf with the developed array and time-efficient pulse sequences, which can potentially provide new insights into vasculature function and metabolic activity in muscles.

## Methods

### ^31^P/^1^H coil design

**Figure 5:**
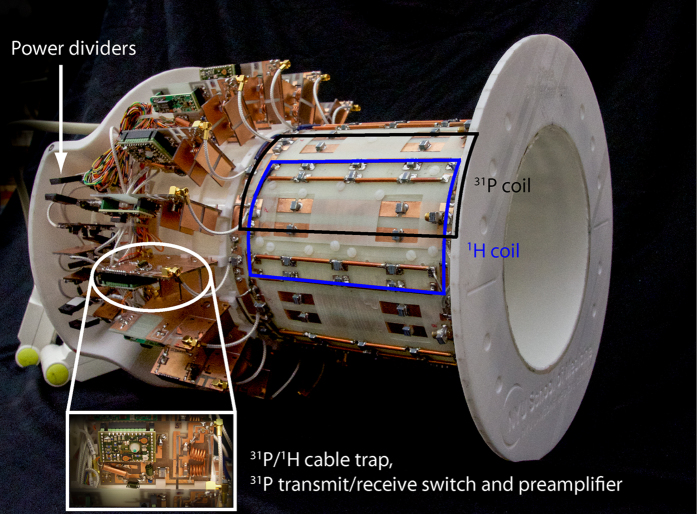
Photograph of the developed ^31^P/^1^H array with the protective cover removed. Overlays highlight an interface board that accommodates the cable trap, transmit/receive switch, and the preamplifier for each coil as well as the power dividers, a ^31^P coil (black), and an ^1^H coil (blue).

**Figure 6:**
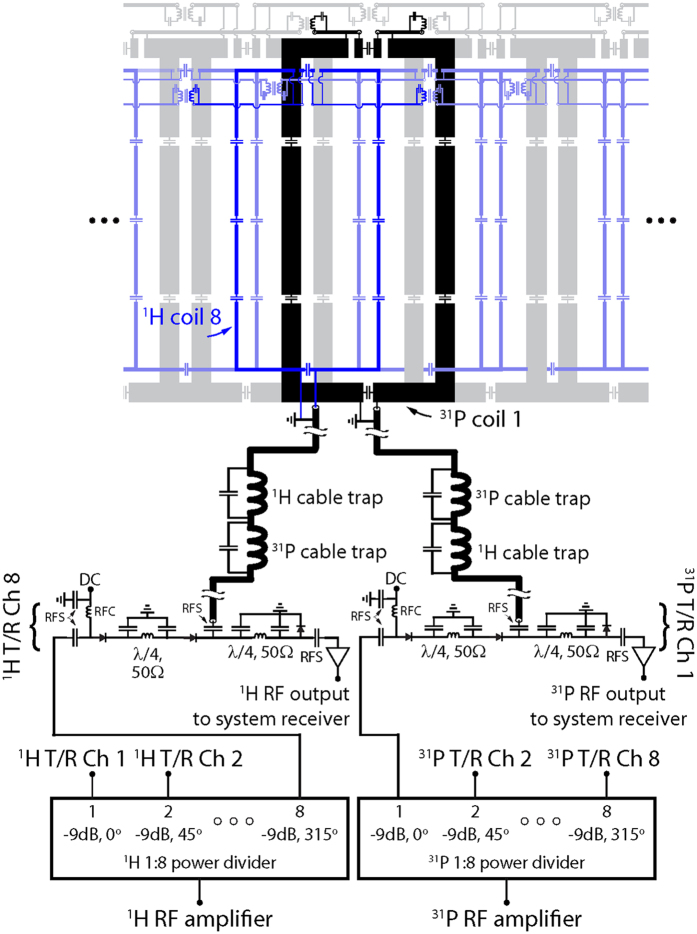
Unrolled schematic diagram of the ^31^P/^1^H coil array and interface. For simplicity, single ^31^P (black) and ^1^H (blue) coils of the 16-channel nested array are highlighted whereas neighboring elements are displayed in a semi-transparent fashion. Abbreviations: RFC = radio frequency choke and RFS = radio frequency short.

The main objective of this project was to construct a ^31^P/^1^H lower extremity coil with a high ^31^P SNR to facilitate dynamic imaging of phosphocreatine kinetics on a 3 Tesla MRI scanner. The secondary objectives included obtaining a uniform ^31^P transmit magnetic field (B_1_^+^) and developing a ^1^H module for anatomical localization, dynamic imaging (i.e. BOLD), and B_0_ shimming. Two (^31^P and ^1^H) transmit/receive arrays were constructed to accomplish these objectives. Both arrays were made up of eight loop coils that encircled the 17-cm-diameter housing structure. The ^1^H array was offset in the azimuthal direction by 22.5° (see Figs 5 and 6) to reduce shielding caused by the ^31^P array. All coils were tuned to 49.9 MHz (^31^P) or 123.2 MHz (^1^H) and matched to 50 Ω while loaded with a water-based gel phantom^41^ with dielectric properties that were designed to mimic muscle tissue^42^. Neighboring and next-nearest coils were decoupled via geometric overlap^43^ and lumped element inductors, respectively.

To generate circularly polarized B_1_^+^ fields, we drove the ^31^P and ^1^H arrays using separate eight-way power splitters that each consisted of three stages of Wilkinson power dividers and quadrature hybrids arranged to provide outputs with 45° phase offsets that corresponded to the azimuthal position of the coils. Individual power splitter outputs were connected to transmit/receive switches to protect the preamplifiers during transmission. The transmit/receive switches utilized a quarter-wavelength-based design similar to a previously outlined method^44^. Individual cable traps were connected to each coil port, and the cable traps were tuned to block common mode current on the coaxial cable shield at ^31^P and ^1^H resonant frequencies. Preamplifier decoupling was accomplished by installing lumped-element phase-shifters at the preamplifier input such that its low input impedance was translated into an inductance that formed a parallel resonant circuit with the coil match capacitor^43^.

### Coil benchmarking

All imaging experiments were performed on a 3 Tesla MRI scanner (Prisma, Siemens Medical Solutions, Erlangen, Germany). The study was fully compliant with the Health Insurance Portability and Accountability Act, and the New York University Institutional Review Board approved the protocol. We scanned human subjects after obtaining their informed written consent. The methods were conducted in accordance with Food and Drug Administration guidelines. We restricted transmit power to 10 W/kg based on MR thermometry measurements using a procedure similar to the method described in ref. 45 to enforce a two-fold safety buffer below the 20 W/kg limit set by the International Electrotechnical Commission (IEC document 60601-2-33 2010).

We performed SNR and B_1_^+^ benchmark measurements with the developed array as well as two commercial coils available at our Center including 1) a dual-nuclei ^31^P/^1^H birdcage knee coil (~19 cm in diameter, Rapid Biomedical, Rimpar, Germany) and 2) a state-of-the-art clinical single-nuclei ^1^H 15-channel knee array (~18 cm in diameter, “TxRx 15Ch Knee Coil”, Quality Electrodynamics, Mayfield, OH, USA). Raw SNR maps were calculated from the signal and noise (with the RF pulse amplitude set to zero) measurements acquired with a gradient echo pulse sequence and processed with the optimal array combination method^43^,^46^. The ^31^P/^1^H imaging parameters were as follows: TR = 10/3.5 s, TE = 6.4/3.6 ms, flip angle = 76/10°, voxel size = 7.8 × 7.8 × 50.0/2.0 × 2.0 × 5.0 mm^3^, and acquisition time = 642/338 s. To account for the spatially dependent ^1^H B_1_^+^, the ^1^H raw SNR maps were scaled according to 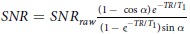, where *α* is the flip angle, TR is the repetition time, and T_1_ = 1.4 s is the relaxation time of the muscle^47^. We calculated the SNR in various muscles by manually segmenting co-registered spin echo anatomical images in the central transverse plane of the coils.

The ^1^H B_1_^+^ field was measured using the method described in ref. 48. Because the pulse sequence was not available for non-proton nuclei, we performed ^31^P B_1_^+^ mapping by scaling the period of a sine curve fit to the pixel-wise signal intensities of a series of gradient echo images that were collected with a range of imaging pulse amplitudes. For both nuclei, the B_1_^+^ mapping resolution was matched to the resolution of the respective SNR measurements.

### Dynamic ^31^P/^1^H-MR experiments

We acquired dynamic ^31^P data using a three-dimensional non-Cartesian fermat looped, orthogonally encoded trajectories (FLORET) sequence^49^,^50^ with a narrowband Gaussian-shaped pulse (duration: 12 ms; bandwidth: 125 Hz) to excite a single metabolite of the ^31^P spectrum (i.e., PCr). The participant performed 180 s plantar flexion exercises at 0.33 Hz according to an acoustic cue on an in-house-developed, MR-compatible ergometer^30^. During exercise, the participant moved the footplate of the ergometer through a 30° range of motion. Resistance was applied by rubber tubing and was set to approximately 40% of the subject’s maximum voluntary contraction (MVC). Images with 6 s temporal resolution were acquired serially before (baseline), during, and after completing the exercise. The acquisition parameters of the FLORET sequence were as follows: TR = 0.5 s, FA = 25°, 3 hubs at 45°, 4 interleaves per hub, 1.7 cm nominal isotropic resolution, 6 s acquisition time per image, and 9 min total acquisition time. The plot in Fig. 3 shows a signal from the gastrocnemius muscle that was manually segmented in a co-registered ^1^H spin echo image.

To measure fat content in the muscle, we applied an ^1^H three-dimensional gradient echo sequence with the following parameters: TE = 2.1, 2.8 and 3.7 ms, flip angle = 3°; TR = 12 ms, FOV = 22 × 22 × 20 cm^3^, acquisition matrix = 128 × 128 × 40, and total acquisition time = 3 min. Water and fat fraction maps were calculated using the Hierarchical IDEAL method^32^.

BOLD dynamics following maximal isometric contractions were measured using an ^1^H one-shot gradient-recalled echo-planar sequence with the following parameters: TR = 1 s, TE = 35 ms, FOV = 25 × 25 cm^2^, slice thickness = 1 cm, acquisition matrix = 64 × 64, and acquisition time = 10 min. We measured the post-contractile response in the soleus muscle, which was characterized by the peak ΔS_max_ and the TTP^51^. The plot in Fig. 4 shows the signal from the soleus muscle that was manually segmented in a co-registered ^1^H spin echo image.

## Additional Information

**How to cite this article**: Brown, R. *et al*. Magnetic Resonance Imaging of Phosphocreatine and Determination of BOLD Kinetics in Lower Extremity Muscles using a Dual-Frequency Coil Array. *Sci. Rep.* **6**, 30568; doi: 10.1038/srep30568 (2016).

## Figures and Tables

**Table 1:** Summary of ^31^P SNR and B_1_^+^ measurements in the developed array and a commercially available dual-nuclei birdcage.

Measurement	^31^P SNR						^31^P B_1_^+^ (nT/V)
ROI	GN(lat)	GN(med)	Soleus	TA	TP	Phantom (center)	Phantom (transverse cross-section)
^31^P/^1^H Array	12.4 ± 2.0	12.1 ± 2.4	8.4 ± 2.2	8.9 ± 1.4	5.6 ± 0.8	8.2	253.4 ± 14.3
^31^P/^1^H Birdcage	5.1 ± 0.7	4.9 ± 0.9	3.8 ± 1.3	4.8 ± 1.3	4.2 ± 1.0	8.0	323.2 ± 6.5

^*^GN = gastrocnemius, TA = tibialis anterior, TP = tibialis posterior, lat = lateral, med = medial. Values are reported as the mean ± standard deviation within the ROI.

**Table 2:** Summary of ^1^H SNR and B_1_^+^ measurements in the developed array and two commercially available coils.

Measurement	^1^H SNR					^1^H B1+ (nT/V)	
ROI	GN(lat)	GN(med)	Soleus	TA	TP	Calf (transverse cross section)	
^31^P/^1^H Array	2.41 ± 0.23	2.52 ± 0.38	1.82 ± 0.24	1.30 ± 0.14	1.28 ± 0.13	89.1 ± 8.8	
^31^P/^1^H Birdcage	1.08 ± 0.14	0.97 ± 0.08	1.14 ± 0.10	0.93 ± 0.11	1.26 ± 0.11	98.7 ± 8.4	
Clinical ^1^H Array	2.47 ± 0.23	2.16 ± 0.28	1.97 ± 0.34	1.45 ± 0.17	1.50 ± 0.16	87.4 ± 6.1	

^*^GN = gastrocnemius, TA = tibialis anterior, TP = tibialis posterior, lat = lateral, med = medial. Values are reported as the mean ± standard deviation within the ROI.
